# Utilisation Trends of Lisdexamfetamine: Insights From Recent Medicine Shortages in Australia

**DOI:** 10.1002/pds.70113

**Published:** 2025-02-11

**Authors:** Jack Janetzki, Lisa Kalisch, Kelly Hall, Nicole Pratt

**Affiliations:** ^1^ Clinical and Health Sciences University of South Australia Adelaide South Australia Australia; ^2^ Quality Use of Medicines and Pharmacy Research Centre University of South Australia Adelaide South Australia Australia

**Keywords:** dispensing, lisdexamfetamine, medicines shortages, trends, utilisation

## Abstract

**Purpose:**

Investigate the impact of recent notified medicine shortages on dispensing patterns of 30, 40, 50, and 60 mg strengths of lisdexamfetamine for treatment of Attention Deficit Hyperactivity Disorder (ADHD) in Australia.

**Methods:**

Pharmaceutical Benefits Scheme (PBS) aggregate dispensing data for 2022–2024 were analysed. Monthly dispensings and Defined Daily Doses (DDDs) for lisdexamfetamine were calculated overall and by product strength.

**Results:**

From January 2022 to August 2023, there was a constant increase in overall dispensing and volume of lisdexamfetamine likely due to expansion of PBS prescribing restrictions allowing subsidy of this medicine for patients 18 years and older in February 2021. Dispensings of the 30 mg strength decreased from August 2023 corresponding with shortages of this product. Dispensings of the 50 mg peaked in October 2023 then decreased. During the shortage of the 30 and 50 mg strengths, dispensings of the 40 and 60 mg strengths increased, however, by December 2023 dispensings of these strengths were also decreasing. Dispensings of 70 mg strengths grew steadily throughout 2024. DDDs changed substantially during the shortage period suggesting that people likely transitioned to different strengths of lisdexamfetamine to maintain their dose.

**Conclusion:**

Dispensing patterns of lisdexamfetamine, by strength, changed significantly during the medicines shortages periods revealing potential changes in prescriber and patient behaviours, such as switching to higher strength products or using medicines intermittently, to maintain continuity of care. To facilitate quality use of medicines during shortages, dispensing patterns must be monitored so that inequities of access can be identified and addressed.

**Summary**

- Monthly dispensings of lisdexamfetamine in Australia have grown consistently from 2022 to 2024, likely due to the expanded subsidy restrictions
- Shortages of lisdexamfetamine have caused fluctuations in dispensings of individual strengths of lisdexamfetamine from late 2023 to early 2024.
- Monthly dispensings of 20, 30, 40, 50 and 60 mg lisdexamfetamine decreased during the shortage.
- Dispensings rates of 70 mg lisdexamfetamine were less affected by shortages.
- Monthly dispensings of 40 mg increased noticeably when other strengths were out of stock potentially due to patients switching to these products; dispensings rapidly declined once other strengths returned to market.

## 1 Introduction

Attention deficit hyperactivity disorder (ADHD) is a chronic condition characterised by persistent inattention, hyperactivity and or impulsivity. ADHD is thought to affect 6%–10% of school‐aged children and is a lifelong condition [1]. Lisdexamfetamine is a first‐line ADHD treatment for patients whose symptoms have stabilised yet desire a psychostimulant with a longer duration of effect [2, 3]. In Australia, lisdexamfetamine is subsidised on the Pharmaceutical Benefits Scheme (PBS), the national medicines formulary [1, 2]. Prior to February 2021, lisdexamfetamine was only subsidised on the PBS when prescribed to children less than 18 years of age, however, since then, PBS restrictions have changed to allow subsidy of lisdexamfetamine to patients over 18 years of age who had not been diagnosed with ADHD or used lisdexamfetamine during childhood [4]. Since 2022, lisdexamfetamine has been the most commonly prescribed medicine for ADHD in Australia [4].

In December of 2023, the drug sponsor for lisdexamfetamine advised the Australian national medicines regulator, the Therapeutic Goods Administration (TGA), of limited availability of 30, 50 and 60 mg strengths of lisdexamfetamine due to manufacturing issues. Shortages of the 40 mg strength were also reported by the sponsor due to an unexpected increase in usage of this product [3, 5]. Shortages of every strength of lisdexamfetamine except 70 mg have been listed in the TGA Medicine Shortage Reports Database [5].

The aim of this study was to describe dispensing patterns of lisdexamfetamine in Australia associated with the reported shortages.

### 1.1 Methodology

PBS Section 85 Date of Supply monthly report data provided by Services Australia were used for this analysis. Data pertaining to supply of all lisdexamfetamine and dexamfetamine strengths and formulations subsidised on the PBS were extracted for the period January 2022 to August 2024 [6]. Total prescriptions dispensed each month were calculated and the percentage change from month to month identified. Monthly trends were calculated for the volume (Defined Daily Dose, DDD) of lisdexamfetamine prescriptions dispensed per 1000 population. The Australia Bureau of Statistics national, state and territory population data estimate for each quarter was utilised as the population denominator [7]. The TGA Medicine Shortage Reports database [5] was searched to identify dates of reported lisdexamfetamine shortages. Microsoft Excel was used for visualisation of the data.

## 2 Results

Monthly dispensings of lisdexamfetamine have increased by 170% from January 2022 (59 982 dispensings in January 2022 compared to 162 178 in August 2024, Figure 1).

**FIGURE 1:**
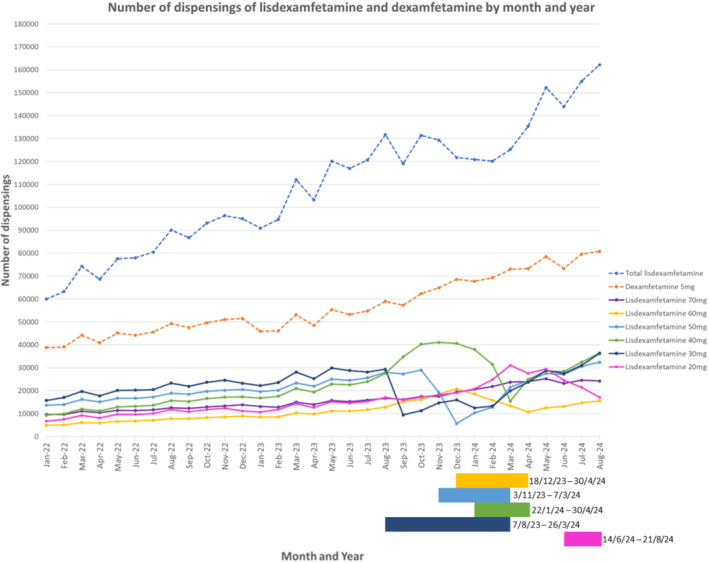
Total dispensings of lisdexamfetamine and dexamfetamine and dispensings of lisdexamfetamine by individual product strength per month from January 2022 to August 2024. Coloured bars represent medicine supply impact dates for each strength listed per Therapeutic Goods Administration Medicines Shortages Database [5].

Dispensing patterns of selected strengths of lisdexamfetamine changed during the medicine shortages periods. A notified shortage of the 30 mg strength from August 7, 2023 to March 26, 2024 saw a 68% reduction in monthly dispensings from August to September 2023 (Figure 1). Similarly, the notified shortage of the 50 mg strength from November 3, 2023 to March 7, 2024 saw monthly dispensings peak at 29029 in October 2023 then reduce to 5663 in December 2023, an 80.5% decrease in dispensings. As these strengths experienced shortage, there was a corresponding increase in monthly dispensings of the 40 mg strength capsules from 27 580 dispensings in August 2023 (when the 30 mg shortage was announced) peaking at 41100 in November 2023 (when the 50 mg shortage was announced), a 49% increase. On December 18, 2023, a shortage of the 60 mg strength was announced by the TGA; monthly dispensings of the 60 mg strength increased until December 2023, peaking at 20818 dispensings, however subsequently declined to 10 766 dispensings in April 2024. Following shortages of the 30, 50 and 60 mg strengths, a shortage of the 40 mg strength was announced (22nd January to 30th April 2024) due to unexpected increase in demand. A rapid decline to 15 433 dispensings in March 2024 was observed as other strengths returned to market and this strength was still in short supply. A shortage of the 20 mg strength occurred towards the end of the study period due to unexpected increase in consumer demand. Total dispensings of dexamfetamine continued to increase during the lisdexamfetamine shortage period. There were no notified shortages of the 70 mg strength during the study period and its use increased throughout the notified shortages periods of other strengths.

During the notified medicine shortage periods, DDDs for the 30 and 50 mg strengths reduced by more than half and the 40 mg strength DDD almost doubled (Table 1). The DDDs for the 20 and 70 mg strength were mostly stable over January 2023 to July 2024 (Table 1) and averaged 0.47 and 1.61 respectively per 1000 population per month suggesting no significant change in population‐level use of these strengths of lisdexamfetamine.

**TABLE 1:** Defined Daily Doses of lisdexamfetamine by strength of capsule per month and percentage change from prior month from January 2023 to August 2024. Orange denotes deviation from usual DDD prior to shortage.

	20 mg	% change	30 mg	% change	40 mg	% change	50 mg	% change	60 mg	% change	70 mg	% change
Jan‐23	0.27	−3.57	0.84	−4.55	0.85	−3.41	1.23	−5.38	0.64	−5.88	1.15	−6.50
Feb‐23	0.30	11.11	0.89	5.95	0.88	3.53	1.27	3.25	0.65	1.56	1.13	−1.74
Mar‐23	0.36	20.00	1.06	19.10	1.06	20.45	1.47	15.75	0.77	18.46	1.32	16.81
Apr‐23	0.32	−11.11	0.95	−10.38	0.97	−8.49	1.38	−6.12	0.74	−3.90	1.22	−7.58
May‐23	0.38	18.75	1.12	17.89	1.15	18.56	1.57	13.77	0.84	13.51	1.38	13.11
Jun‐23	0.37	−2.63	1.08	−3.57	1.13	−1.74	1.54	−1.91	0.83	−1.19	1.34	−2.90
Jul‐23	0.38	2.70	1.05	−2.78	1.19	5.31	1.59	3.25	0.87	4.82	1.38	2.99
Aug‐23	0.43	13.16	1.09	3.81	1.37	15.13	1.74	9.43	0.96	10.34	1.45	5.07
Sep‐23	0.40	−6.98	0.35	−67.89	1.73	26.28	1.70	−2.30	1.13	17.71	1.41	−2.76
Oct‐23	0.43	7.50	0.42	20.00	1.99	15.03	1.79	5.29	1.20	6.19	1.51	7.09
Nov‐23	0.45	4.65	0.55	30.95	2.03	2.01	1.19	−33.52	1.37	14.17	1.52	0.66
Dec‐23	0.47	4.44	0.59	7.27	2.01	−0.99	0.35	−70.59	1.54	12.41	1.68	10.53
Jan‐24	0.51	8.51	0.46	−22.03	1.87	−6.97	0.64	82.86	1.37	−11.04	1.78	5.95
Feb‐24	0.61	19.61	0.49	6.52	1.55	−17.11	0.79	23.44	1.17	−14.60	1.88	5.62
Mar‐24	0.76	24.59	0.74	51.02	0.76	−50.97	1.32	67.09	0.99	−15.38	2.04	8.51
Apr‐24	0.68	−10.53	0.87	17.57	1.23	61.84	1.50	13.64	0.79	−20.20	2.07	1.47
May‐24	0.73	7.35	1.07	22.99	1.40	13.82	1.69	12.67	0.93	17.72	2.17	4.83
Jun‐24	0.60	−17.81	1.01	−5.61	1.40	0.00	1.67	−1.18	0.97	4.30	1.99	−8.29
Jul‐24	0.53	−11.67	1.15	13.86	1.60	14.29	1.88	12.57	1.08	11.34	2.12	6.53
Aug‐24	0.42	−20.75	1.34	16.52	1.79	11.87	1.99	5.85	1.15	6.48	2.09	−1.42

## 3 Discussion

Monthly dispensings of lisdexamfetamine for treatment of ADHD have continued to increase since the expansion of the PBS criteria in February 2021 and despite recent medicine shortages [4]. Increasing demand for lisdexamfetamine throughout the study period and medicine shortages both significantly impacted dispensing rates of 30, 40, 50 and 60 mg strengths of lisdexamfetamine in Australia towards the end of 2023 and throughout 2024. Despite medicine shortages, the overall volume of use of lisdexamfetamine continued to increase, however, the composition of the dispensings according to the strength of product dispensed varied.

Drug sponsors are required to alert national medicine regulators to shortages as soon as the sponsor is aware that supply may be impacted. The notification of lisdexamfetamine shortages to the TGA was made in December 2023, however, a decline in dispensings of the 30 and 50 mg strengths is observed prior to this date; in August and November respectively. Corresponding to the notified shortage for the 30 mg product, dispensing volumes of the 30 mg product decreased and dispensing volumes of the 40 mg product doubled. When a shortage was announced for the 50 mg product, the volume of dispensing decreased, and the volume dispensed of the 60 mg product increased. Once the 50 mg strength came back into stock, dispensings of the 60 mg declined. This suggests that medicines shortages can have several impacts on patient use behaviour. Several plausible actions could explain the observed dispensing patterns: patients may use available capsules sparingly (reduced DDD), resort to dispersing higher strength capsules in liquid to achieve required daily dose (increased DDD) or doubling dispensings to achieve higher doses. The sustained elevated DDD of 1.61 for the 70 mg strength is concerning as the maximum daily dose of lisdexamfetamine is 70 mg, however it is possible that a proportion of patients were having this strength dispensed but dispersing the capsule to take an appropriate dose to achieve desired symptom control [8]. Some patients could also be accessing more than one strength of lisdexamfetamine to achieve their required daily dose. Although the number of dexamfetamine prescriptions dispensed each month increased over the study period, the trend remained relatively stable suggesting that it is unlikely that a large number of patients switched from lisdexamfetamine to dexamfetamine during periods of lisdexamfetamine shortage; however, individual patient level data is required to confirm this. Changing medications or making alternative arrangements (e.g., dispersing capsules) is challenging and may be associated with patient confusion, suboptimal control of symptoms or emergence of adverse effects due to under‐dosing or over‐treatment respectively, compromising patient safety and treatment efficacy [9]. Frequent interruptions to supply may increase out‐of‐pocket costs associated with additional health care visits and being required to purchase alternative strengths or different medicines to maintain symptom control.

There are currently several strategies to mitigate impact of medicines shortages in Australia namely advanced alerts to health professionals about anticipated shortages, provision of the searchable medicines shortages database by the TGA, provision to prescribe registered or unregistered medicines from overseas, substitution instruments or personal importation provisions [10]. Such strategies may reduce the impact of medicine shortages, however often shortages are noticed at a pharmacy level before regulators notify the public and such strategies can only be implemented once a shortage is realised. Monitoring aggregate dispensings of medicines in real‐time is one strategy to identify medicine shortages in a timely manner. Our analysis demonstrates that rapid changes to dispensing volumes could be an early indicator of shortages particularly where patients are using individualised dosing regimens that may require multiple strengths of capsules to achieve symptom control. This was observed in the case of the 30 mg and 50 mg strengths where DDDs decreased from 1.09 (August 2023) to 0.35 (September 2023) and from 1.19 (November 2023) to 0.35 (December 2023), respectively. An increase in DDD was also observed for the 40 mg strength of lisdexamfetamine from 1.37 (August 2023) to 1.73 (September 2023) indicating a change in population‐level dispensing patterns and behaviours.

Despite the shortage of lisdexamfetamine being resolved as of April 30th according to the drug sponsor and the TGA [11], pharmacists anecdotally reported that in the months following the shortage, warehouses still had limited or no stock to send to pharmacies. Subsequently, in June 2024, the TGA were notified by the sponsor that 20 and 60 mg strengths of lisdexamfetamine were experiencing shortage [3, 5].

While the use of aggregate dispensing data in this study is able to demonstrate population‐level changes in dispensing trends, investigation of treatment patterns including switching between medicines in a person‐level dataset such as the PBS 10% extract would allow for additional recommendations for people in priority groups particularly during shortages. Real‐time monitoring of national dispensings patterns could help ensure that medicine shortage policies are implemented until dispensing rates return to normal.

## 4 Conclusion

This analysis provides insights into the utilisation of lisdexamfetamine prior to, during and after medication shortages. In the context of increasing use of lisdexamfetamine due to expanded PBS subsidy restrictions, there was increased demand and overall utilisation of lisdexamfetamine in Australia. Despite medicine shortages in late 2023, patients appeared to stay on treatment; however, they responded by changing the products they were using. Monitoring dispensings patterns and fluctuations, particularly DDDs during shortages, can help to ensure access to medicines is maintained. Such monitoring will help to inform and promote early implementation of medicine shortage policies to enable equitable access to medicines when supply is uncertain and identify areas of concern such as increased risk of adverse effects to patients.

### 4.1 Plain Language Summary

In this study, we examined how shortages of certain strengths of lisdexamfetamine, a medication for Attention Deficit Hyperactivity Disorder, in Australia, influenced how it was prescribed and used by patients. We looked at data from the Pharmaceutical Benefits Scheme (PBS) between 2022 and 2024 and found that overall dispensing of lisdexamfetamine increased steadily during the study period, likely due to changes in PBS rules allowing adults to receive the medication. During the study period, however, we show that the 30, 40. 50 and 60 mg strengths were affected by medicines shortages. When these strengths were in shortages, people seemed to switch to other strengths to maintain treatment. From these observations, were suspect that patients and doctors adjusted their behaviours to minimise impact on the person and their condition. Some people may have been dispensed higher strengths or changed how frequently they used the medicine. Monitoring dispensing patterns is crucial to ensure fair access to medication during shortages and to understand how best to manage such situations in the future.

## Ethics Statement

The PBS Section 85 Date of Supply data is freely available via the PBS website. As aggregate level prescription and expenditure information ethics approval was not required.

## Conflicts of Interest

NP is a member of Drug Utilisation Sub Committee (DUSC) of the Pharmaceutical Benefits Advisory Committee (PBAC). The other authors declare no conflicts of interest.

## Data Availability

Data for this study is freely accessible via The Pharmaceutical Benefits Scheme website: https://www.pbs.gov.au/info/browse/statistics.
